# Toward a Harmonized and Standardized Protocol for the Determination of Total Hydroxytyrosol and Tyrosol Content in Virgin Olive Oil (VOO). The Pros of a Fit for the Purpose Ultra High Performance Liquid Chromatography (UHPLC) Procedure

**DOI:** 10.3390/molecules24132429

**Published:** 2019-07-02

**Authors:** Maria Z. Tsimidou, Nikolaos Nenadis, Aspasia Mastralexi, Maurizio Servili, Bojan Butinar, Stefania Vichi, Ole Winkelmann, Diego Luis García-González, Tullia Gallina Toschi

**Affiliations:** 1 Laboratory of Food Chemistry and Technology, School of Chemistry, Aristotle University of Thessaloniki (AUTH), 541 24 Thessaloniki, Greece; 2 Department of Agricultural, Food and Environmental Sciences, University of Perugia, (UNIPG), Via San Costanzo s.n.c., 06126 Perugia, Italy; 3 Laboratory of the Institute for Oliveculture, Science and Research Centre Koper (ZRS), Zelena ulica 8k, SI-6310 Izola, Slovenia; 4 Department de Nutrició, Ciències de l’Alimentació i Gastronomia, XaRTA, INSA-UB, Campus De l’Alimentació Torribera, Universitat de Barcelona (UB), Av. Prat de la Riba, 171, 08028 Santa Coloma de Gramenet, Spain; 5 Eurofins Analytik GmbH, Neuländer Kamp 1, 21079 Hamburg, Germany; 6 Instituto de la Grasa (CSIC), Ctra. De Utrera, km. 1, Campus Universitario Pablo de Olavide—Building 46, 41013 Seville, Spain; 7 Department of Agricultural and Food Sciences, Alma Mater Studiorum, University of Bologna (UNIBO), Piazza Goidanich, 60, I-47521 Cesena (FC), Bologna, Italy

**Keywords:** virgin olive oil, hydroxytyrosol, tyrosol, health claim, European Commission Regulation 432/2012, standardization, UHPLC-DAD, HPLC-DAD, LC-HRMS, ^1^H-NMR spectroscopy

## Abstract

Τoward a harmonized and standardized procedure for the determination of total hydroxytyrosol and tyrosol content in virgin olive oil (VOO), the pros of a recently published in house validated ultra high performance liquid chromatography (UHPLC) protocol are discussed comparatively with those of other procedures that determine directly or indirectly the compounds hosted under the health claim on “olive oil polyphenols” (EC regulation 432/2012). Authentic VOOs were analyzed with five different liquid chromatographic separation protocols and ^1^H-NMR one in five different laboratories with expertise in VOO phenol analysis within three months. Data comparison indicated differences in absolute values. Method comparison using appropriate tools (Passing-Bablok regression and Bland Altman analyses) for all protocols vs. the UHPLC one indicated slight or statistically significant differences. The results were also discussed in terms of cost effectiveness, detection means, standard requirements and ways to calculate the total hydroxytyrosol and tyrosol content. Findings point out that the in-house validated fit for the purpose UHPLC protocol presents certain pros that should be exploited by the interested parties. These are the simplicity of sample preparation, fast elution time that increase the number of samples analyzed per day and integration of well-resolved peaks with the aid of only two commercially available external standards. Importance of correction factors in the calculations is stressed.

## 1. Introduction

The interest in the determination of phenolic compounds in virgin olive oil (VOO) was initially related with their contribution to the oil stability in the dark [1] and soon after with their effect to the sensory attributes of the oil [2]. The colorimetric estimation of the total phenol content using the Folin-Ciocalteu reagent was a good compromise for both objectives. However, no harmonized approach can be found in the literature mainly in terms of results expression though it has been repeatedly stressed that the choice of standards influences quantitative results [3,4]. Due to a broader interest in the antioxidant activity of natural phenolic antioxidants since the early 90s, separation approaches were developed for the identification and quantification of the individual phenolic and related compounds extracted in the polar fraction of VOO using aqueous methanol mixtures of varying composition [5]. The developed procedures reflected the expertise of the scientists involved and were mainly gas chromatographic and liquid chromatographic ones [6,7]. The second category of analytical methods prevailed with the advance of detection systems and mass spectrometry (MS) coupling. The instrumental advances improved identification of the partially resolved complex forms of the oleuropein and ligstroside derivatives, which are transferred in VOO during olive processing [8]. The almost universal interest in these phenolic compounds, which are mainly found in the fresh high quality extra VOOs, explains the publication of hundreds of papers that give data on their concentrations regarding the effect of biotic and abiotic factors, processing and storage. In 2009, the International Olive Council (IOC) adopted an HPLC method for the ‘determination of biophenols in olive oils by HPLC’ that was based on the isolation of polar fraction with 80:20 *v*/*v* methanol:water and quantification at 280 nm via converting the sum of the areas of the related chromatographic peaks to tyrosol equivalents. The latter is achieved using the corresponding relative response factor of tyrosol to that of syringic acid used as internal standard [9] (IOC). The method contains some elements of validation but no published paper provides in house validation data that support the standard operation procedures applied in the ring test. No HPLC protocols published before or after the IOC method offer some dramatically improved separation of individual compounds or are fully validated to our knowledge.

Approval of the health claim on ‘olive oil polyphenols’ regarding ‘protection of blood lipids from oxidative stress’ raised analytical concerns for the determination of the 5 mg of hydroxytyrosol and its derivatives (e.g., oleuropein complex and tyrosol) per 20 g of olive oil [10,11,12]. It is important that IOC recognized that the existing procedure cannot support the new analytical need as it is [13]. The health claim on the phenolic compounds of olive oil of the EU Regulation 432/2012 is backed by a European Food Safety Authority (EFSA) scientific opinion [14], which did not propose a specific analytical procedure for the determination of the responsible compounds. This fact together with unclear terminology had a negative effect to the commercial exploitation of this claim. Recently, it was scientifically justified [12] why all the forms of hydroxytyrosol (Htyr) and tyrosol (Tyr) found in the oil should be considered to support the claim requirement.

After the launching of both the respective EFSA opinion and the EC Regulation 432/2012 many papers aimed at determining the amount of at least 5 mg bioactive phenols/20 g oil [10,15,16,17,18,19,20]. These papers indicated the preference among scientists to simplify the profile of phenolic compounds through hydrolysis of the bound forms. Since the efforts of the scientists continue to different directions in terms of analytical strategies, there is a need for consensus among all interested parties that will lead in a harmonized and standardized protocol suitable for commercial needs. In the frame of the OLEUM project, seeking for the harmonization of procedures that can then be adopted in the analysis of olive oil for both regulatory and research purposes [21] (http://www.oleumproject.eu/), a ‘fit for the purpose ultra high performance liquid chromatography (UHPLC) protocol for the determination of the total content of Htyr and Tyr’ [22], was in house validated and found to be satisfactory in terms of performance criteria. The content of the two phenols was determined in the polar fraction per se and in its hydrolysate using two standard curves at 280 nm.

The present collaborative work highlights the specific pros of this published in house UHPLC protocol that deserve further evaluation by organizations such as Association of Analytical Communities (AOAC), IOC, European Union (EU) toward a harmonized and standardized procedure that will address the analytical challenges raised by the respective health claim, in a way that will not cause disputes upon implementation in all types of laboratories (industrial or official quality control laboratories) and the results can be produced by widely available technology. To this view a number of authentic VOOs were analyzed with the abovementioned method and the results are discussed in comparison to those produced by experts for the same samples using different variations of the IOC HPLC procedure. The results are discussed in terms of cost effectiveness, detection means, standard requirements and ways to calculate the total content of Htyr and Tyr. Moreover, the results of the UHPLC approach are also compared with those obtained by means of ^1^H-NMR spectroscopy. NMR spectroscopy procedures are based on a different principle and are also examined by the scientific community for the same purpose [23].

## 2. Results and Discussion

### 2.1. Expertise of Laboratories 1–5

Laboratories 1–4 have a long expertise in olive oil analysis and in the chemistry and analysis of polar phenols as it is proven by their published work and participation in national, European and international committees for olive oil. Laboratory 1 developed and validated protocol A and performed analyses using also HPLC conditions (protocol B) according to its past expertise. Laboratory 2 is accredited to carry out the HPLC analysis of polar phenols according to IOC method (protocol C). Laboratory 3 (protocol D) has the longest experience in phenol analysis and characterization in Europe. Laboratory 4 (protocol E) has expertise in gas chromatography–mass spectrometry (GC–MS) and LC–MS analyses of volatile and non-volatile compounds of olive oil. Laboratory 5 (protocol F), with a specialization to NMR spectroscopy and particularly the NMR analysis of olive oils, is accredited in the field of quantitative NMR spectroscopy and belongs to a wider group of laboratories forming a company that is a world leader in food product testing.

### 2.2. Liquid Chromatographic Analytical Protocols

#### 2.2.1. Protocols Involving Acidic Hydrolysis of the Polar Fraction (PF)

**Figure 1:**
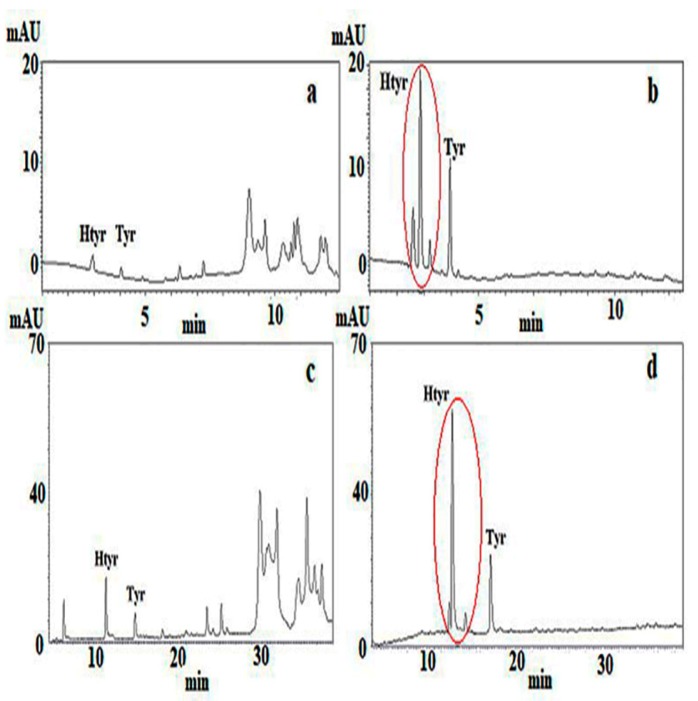
Profile at 280 nm of S-26 VOO polar fraction (PF) prior (**a**,**c**) and after (**b**,**d**) acidic hydrolysis using protocol A (**a**,**b**) and protocol B (**c**,**d**).

Protocols A and B involved determination of Htyr and Tyr in the polar fraction (PF) before and after acidic hydrolysis of all the Htyr and Tyr bound forms and were performed in Laboratory 1 by the same analysts. The target analytes were, thus, determined through integration of well-resolved peaks and with the aid of commercially available external standards at 280 nm. Extraction of the polar fraction under protocol A was as described in the IOC method (methanol:water, 80:20, *v*/*v*) without the addition of internal standard, whereas under protocol B, extraction was performed using methanol:water, 60:40, *v*/*v*, vortexing (2 min) and centrifugation (3500 rpm, 10 min). The results, shown in Table 1 and Table 2, were calculated in the same way taking into account correction for mass difference between free and bound forms and are expressed as the sum of total Htyr and Tyr rounded to the first integer. In Figure 1a–d chromatograms before and after hydrolysis of the PF are also shown. Using protocol A the time needed for sample preparation involving isolation of the PF according to IOC [9] and acidic hydrolysis as proposed by Mulinacci et al. [24] was ~1.2-fold higher than that proposed by Mastralexi et al. [10] (protocol B) but ~2.2-fold lower than that needed for the direct hydrolysis of phenols in the oil according to Romero and Brenes [15]. Elution time using the UHPLC protocol was the ~1/3 of that needed using HPLC conditions (IOC elution protocol). Overall, except for the unequivocal assignment of peaks to target analytes, application of protocol A led to an increase in the number of samples analyzed per day and a significantly lower solvent consumption. The total Htyr and Tyr content ranged from 0 up to ~12 mg/20 g of oil. Frequency distribution of the rounded values revealed that 16 out of the 30 samples addressed the limit of ≥ 5 mg/20 g oil. It should be stated that if correction factors as proposed would not be taken into account the corresponding range would be 0–5 mg/20 g oil and only one sample, namely S-26 would satisfy the legal limit. Applying protocol B the same samples except for two (S-10, S-12) were found with a content ≥5 mg/20 g oil. It can be said that in case PF hydrolysis is applied the major pro of protocol A is the speed of analysis compared to HPLC conditions.

#### 2.2.2. Protocols Not Involving Acidic Hydrolysis of the PF

**Figure 2:**
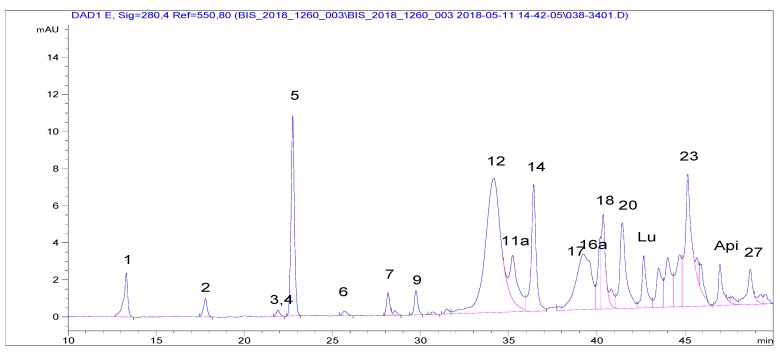
Profile at 280 nm of S-26 VOO PF using UHPLC-diode array detection (DAD; protocol C; 1: Htyr, 2: Tyr, 3: Vanillic acid, 4: Caffeic acid, 5: Syringic acid (IS), 6: Vanillin, 7: *p*-coumaric acid, 9: Ferulic acid, 11a: Decarboxymethyl oleuropein aglycone, oxidized dialdehyde form, 12: Decarboxymethyl oleuropein aglycone, dialdehyde form, 14: Oleuropein aglycone, dialdehyde form, 16a: Decarboxymethyl ligstroside aglycone, oxidized dialdehyde form 17: Decarboxymethyl ligstroside aglycone, dialdehyde form, 18: Pinoresinol, 1-acetoxy-pinoresinol; 20: Ligstroside aglycone, dialdehyde form, Lu: Luteolin, 23: Oleuropein aglycone, aldehyde and hydroxylic form, Api: Apigenin, 27: Ligstroside aglycone, aldehyde and hydroxylic form).

Laboratories 2–4 extracted VOO PFs by the IOC recommended procedure. The accredited Laboratory 2 applied the IOC elution conditions (protocol C), therefore, the corresponding run was three-fold longer than that of protocol A (Figure 2). Peak resolution was better than that according to the IOC HPLC conditions (Figure 1c) that can be assigned to a certain extent to the fact that a more advanced packing material than the recommended one by IOC was used. This material had the same pore size (80 Å) and a surface area two-fold higher and 19% carbon load instead of 11.5. The compounds had been identified at the setup of the method by LC–MS and then upon application in terms of relative retention time to that of the internal standard, syringic acid. Only the peaks assigned to Htyr, Tyr and their bound forms, including four oxidized ones, were integrated. Using a second detection means (LC–MS (TQd)) some additional peaks were assigned as Htyr and Tyr bound forms. It is pointed out that quantification assumes that all compounds present the same response toward the internal standard. Results are shown in Table 3.

Using protocol C, only half of the samples that satisfied the limit using protocol A were found to contain ≥5 mg/20 g oil. These samples (S-1, S-3, S-4, S-15, S-17, S-24, S-26), also satisfied the required limit with protocol B. Inclusion of additional peaks in the calculation affected marginally the number of samples satisfying the minimum required amount (Table S1). The pros of protocols A and B involving PF hydrolysis over the IOC procedure is obvious and in line with the decision of this body to search for a fit for the purpose method. Except of speed, protocols A and B provide results without the need of MS detection even at the step of method introduction in a laboratory.

**Figure 3:**
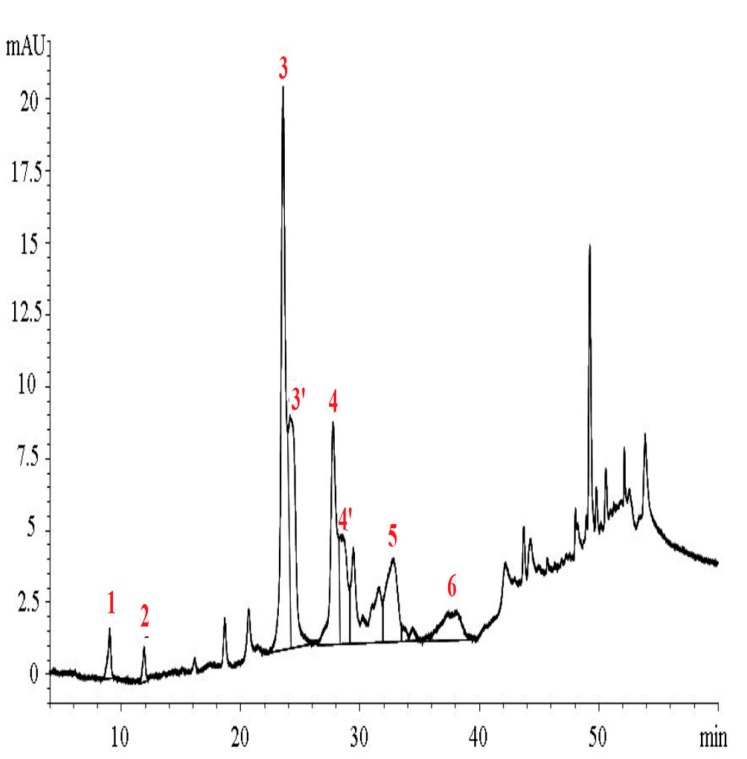
Profile at 278 nm of S-26 VOO PF using HPLC-DAD (protocol D) (peak numbers: 1: Htyr, 2: Tyr, 3: Decarboxymethyl oleuropein aglycone, dialdehyde form, 3′: Other structure of decarboxymethyl oleuropein aglycone, dialdehyde form, 4: Decarboxymethyl ligstroside aglycone, dialdehyde form, 4′: Other structure decarboxymethyl ligstroside aglycone, dialdehyde form, 5: Oleuropein aglycone, 6: Ligstroside aglycone).

Protocol D (Laboratory 3) involved a gradient elution system of 73 min (~2.4-fold longer than that of protocol A) of a binary mixture (acidified water and methanol) instead of the ternary one (acidified H_2_O, methanol, acetonitrile) proposed in the IOC protocol. The column packing material used (ODS-1) though of the same pore size and surface area had a carbon load of 6.2% instead of the 11.5% of the ODS-2 recommended one. This issue, plus the fact of artifact formation due to the increased amount of methanol in the elution system may partially explain the poorer resolution pattern of the chromatogram of Figure 3 in relation to that of Figure 2 or even of Figure 1c for the same VOO sample. The latter was obtained using an end-capped Nucleosil ODS with a 1.6-fold higher surface than that of ODS-1 and a carbon load of 15%. According to protocol D calculation of total Htyr and Tyr content was based on the quantification of six compounds using external standards for five of them, which were either commercially available or laboratory isolated and the response factor of oleocanthal for ligstroside aglycone. In this case no need for mass correction is needed, the mg of the six compounds were summed up to give the final content of all Htyr and Tyr forms.

As evidenced, peaks corresponding to bound forms of Htyr and Tyr are not well resolved making difficult their integration and consequently accurate quantification for the purpose of the health claim. Formation of artifacts due to interaction with methanol might add difficulty to quantification of compounds 3 and 4. Data for the total Htyr and Tyr content delivered according to Laboratory 3 expertise are given in Table 4.

This protocol produced higher values than those using protocol A for 23 samples, so that 21 out of the 30 fulfilled the desirable amount of 5 mg/20g oil. From these samples, five were found of the same content as with protocol A, nine (S-4, S-5, S-10, S-12, S-16, S-19, S-28, S-29, S-30) were found to contain slightly higher content by 1 mg/20 g oil, whereas in 14 (S-1, S-3, S-6–S-9, S-11, S-14, S-15, S-17, S-18, S-24–S-26) the difference was in the range of 2 to 7 mg/20 g oil. Findings need further investigation taking into account that only six compounds were determined. Protocol A seemed to retain the pros of speed and less sophistication in standards used (two external curves instead of five ones and a response factor).

**Figure 4:**
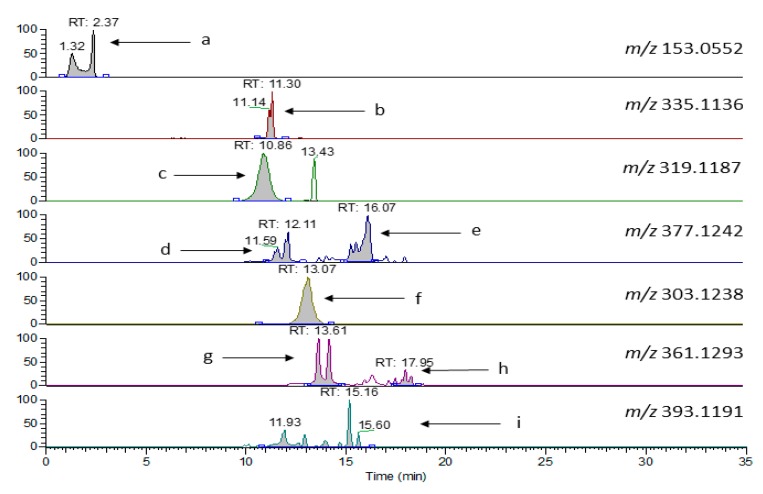
Extracted ion chromatograms in negative ionization mode (protocol E) for molecular ions of a: Htyr, b: Decarboxymethyl oleuropein aglycone oxidized dialdehyde form, c: Decarboxymethyl oleuropein aglycone, d: Oleuropein aglycone dialdehyde form (sum of isomers), e: Oleuropein aglycone aldehyde and hydroxylic form (sum of isomers), f: Decarboxymethyl ligstroside aglycone, g: Ligstroside aglycone dialdehyde form (sum of isomers), h: Ligstroside aglycone aldehyde and hydroxylic form (sum of isomers), i: Oleuropein aglycone oxidized aldehyde and hydroxylic form (sum of isomers) in S-26 VOO PF by liquid chromatography-heated electrospray ionization-high resolution mass spectrometry (LC-HESI-HRMS).

Laboratory 4 (protocol E) exploited the ability of MS to determine specific analytes with no influence from overlapping signals under UV detection mode. A short (10 cm) fused core column, performing in between of UHPLC and conventional HPLC capability, was used. Focus was on major Htyr and Tyr bound forms, their isomers and their oxidation products (Figure 4). As evidenced the total run lasts 35 min, close to that for PF analysis before hydrolysis using protocol A.

Quantification was then based on the assumption that the response factor for all compounds relative to *o*-coumaric acid used as the internal standard was 1, a compromise that introduces some bias in the quantified levels considering the findings of Mateos et al. [25] on olive oil phenol response factors relatively to used internal standards (ISs) under LC–MS conditions of analysis. Additionally, the concentration determined for the aldehyde derivatives should be lower than expected due to artifact formation with methanol and water, used as solvents for the elution in HPLC [26], since these artifacts would have a different molecular ion than those depicted in Figure 4. The quantitative results are provided in Table 5.

As evidenced, 12 samples were found with a content of total Htyr and Tyr ≥ 5 mg/20 g oil of which the 11 were those found with protocols A and B. Samples S-14 and S-18 were found to contain 5 mg instead for 4 mg using protocol A. The pro of protocol A is cost effectiveness, including detection means requirement, and simplicity in approach.

#### 2.2.3. Data Comparison

Regarding the content of total Htyr and Tyr (free and bound forms) obtained for the same samples using the A-E liquid separation protocols (Table 1, Table 2, Table 3, Table 4 and Table 5), it is clear that there are differences in absolute values but there may be a good agreement in terms of statistical comparisons. Pearson correlation analysis is a typical statistical approach to compare different methods. However, in literature there is disagreement whether such correlation provides accurate information for method comparison. Researchers argue that it cannot show whether there is a constant or proportional difference between methods. In addition, it is sensitive to data distribution and outliers and assumes no error in the measurement [27]. Therefore, alternative approaches were sought as more appropriate. For example, comparison using regression lines is recommended when methods are to be compared over a range of different analyte concentrations [28]. Passing-Bablok regression analysis is appropriate for such a task and is widely used in the field of clinical chemistry. This treatment allows estimation of method agreement and detection of possible bias between them. It is considered as robust, non-parametric and non-sensitive to the distribution of errors and data outliers. The regression equation via intercept and slope values provides information on constant and proportional measurement error and in combination with calculated confidence limits conclusion on agreement of measurements can be made [27]. The derived data showed that there was equality of measurements only between protocols A and B because the criteria set for slope and intercept according to the calculated confidence limits were met (Table S2). Bland Altman analysis is a more recent statistical tool. It examines the agreement between two analytical methods by studying the mean difference in measurements for a set of samples and constructing limits of agreement. The ‘difference plot’ permits to evaluate distance of measurements from zero (bias) and its variability (error). Interpretation of the calculated limits of agreement relies on scientists, who accept them or not [29]. Examination of ‘difference plots’ (Figures S1–S5) of protocol A values compared pairwise with the values of the other chromatographic protocols provided the following mean difference (bias) values (mg/20 g oil): −0.67 (B), −2.07 (C), −1.33 (C’), 1.93 (D) and −1.0 (E). Thus, the closer values were those obtained by protocol B. The negative sign indicated that protocol A afforded higher values than the other protocols with the exception of protocol D. The calculated mean difference values indicated that the smallest one was between protocols A and B. Such a value was rather low, justifying, thus, the equality between the two protocols found with Passing-Bablok regression analysis. On the other hand, the mean difference value in the range −1.0 to 1.93 (mg/20 g oil) explained the conclusion drawn from the Passing-Bablok regression analysis that protocols C (C’), D and E differed in performance from A.

### 2.3. ^1^H-NMR Spectroscopy Approach

**Figure 5:**
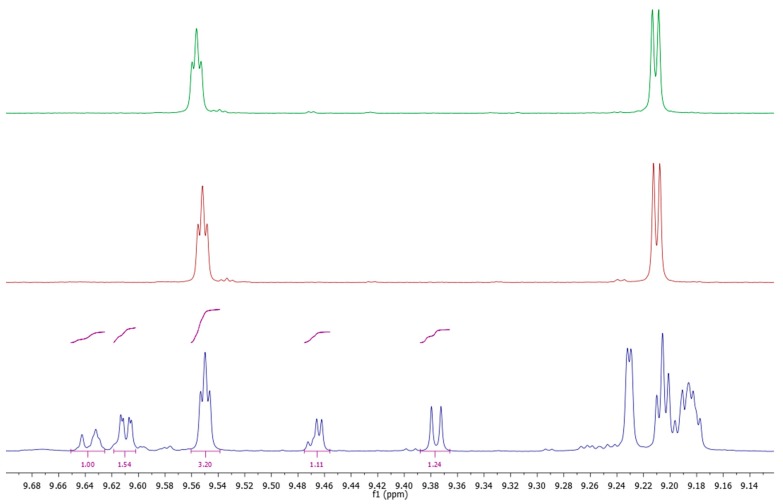
^1^H-NMR spectra (400 MHz, CD_3_CN, aldehyde region) from top to bottom: Decarboxymethyl oleuropein aglycone dialdehyde form (oleacein), decarboxymethyl ligstroside aglycone dialdehyde form (oleocanthal), sample S-26.

Protocol F is based on a completely different principle and it does not involve chromatography or hydrolysis steps. Sample preparation is done via extraction of the oil with acetonitrile and direct subsequent ^1^H-NMR analysis of the polar fraction. The aldehydic region of the NMR spectrum is used to quantify the different bound forms of Htyr and Tyr, analogous to a previously described method [23] with some modifications. The profile of the aldehydic region of VOO S-26, as well as those of pure decarboxymethyl oleuropein aglycone dialdehyde form (oleacein), and decarboxymethyl ligstroside aglycone (oleocanthal) are given in Figure 5. The determined levels are presented in Table 6. As the ^1^H-NMR signals of the respective bound forms of Htyr and Tyr partly overlap in acetonitrile it was not easily possible to discriminate between the two as in the other protocols, and only the sum of Htyr and Tyr bound forms was determined.

The VOO samples satisfying the minimum concentration limit were 22 out of 30, that is six more than those found with protocol A, although a limited number of compounds was considered.

Comparison of the values of protocol A with those produced by protocol F using Passing-Bablok regression showed that there was no equality of measurements since only for the estimated slope (1.113) the value 1 fell within the upper (1.248) and lower (0.9775) confidence interval values and not for the intercept (upper confidence interval value: 1.99, lower confidence interval value: 0.3659). Bland Altman analysis (Figure S6) showed that only for three samples the measurement difference was zero, whereas for the rest of the samples was ≥ 1 mg/20 g oil. As a matter of fact, protocol F was found to produce values with a positive mean difference value of 1.77 mg/20 g oil.

### 2.4. The Pros of the Fit for Purpose Protocol A

Overall evaluation of the findings of the present study points out that the in-house validated UHPLC protocol A presents certain pros that should be taken into account for further consideration by the official bodies toward a consensus for a harmonized and standardized method fit for the purpose of the health claim. Summarizing, these are the simplicity of sample preparation, the fast elution time that may increase the samples analyzed per day and the integration of well-resolved peaks with the aid of only two commercially available external standards at 280 nm taking into account appropriate correction factors in the calculations. UHPLC instrumentation is expected to replace slowly but steadily the HPLC ones in food analytical laboratories as it is observed in other disciplines. Recently, UHPLC approaches have been applied to olive oil analysis [30,31]. Taking into account that the capital cost difference is rather small (~10.000 €), the increase in productivity may make possible the replacement of ~2–3 HPLC systems by one UHPLC [32]. The capital investment for a UHPLC is many folds lower than that for a NMR spectrometer such as the one used the present study. In case protocol A is selected for full validation samples from different cultivars/origin should be also analyzed as it is a common practice.

## 3. Materials and Methods

### 3.1. VOO Samples

The VOO samples examined (S-1–S-30) were obtained at AUTH (Laboratory 1) directly from various producers and locations. All samples were of the 2017–2018 crop season and satisfied the criteria of extra VOO category [33]. Appropriate aliquots of each one were transferred in amber glass bottles, sealed and dispatched to collaborating Laboratories 2–5. Upon reception, the bottles were stored in the dark at −18 °C. Analyses were carried out as described in the following paragraphs in all laboratories involved within a three-month period.

### 3.2. Chemicals, Instrumentation and Methods

Due to the collaborative character of the study, information is briefly given in the text per laboratory. In all laboratories extraction of the polar fraction was according to the IOC protocol unless otherwise stated. Each VOO was extracted in triplicate, the three extracts were combined to form a representative one, which was then analyzed in triplicate.

#### 3.2.1. Laboratory 1

##### Protocol A

Chemicals, UHPLC instrumentation, extraction, hydrolysis conditions, elution, dentification and quantification of Htyr and Tyr were according to Tsimidou et al. [22]. Elution was carried out on a 75 mm × 2.0 mm, 1.6 μm Shim-pack XR-ODS III Shimadzu (Kyoto, Japan).

##### Protocol B

Chemicals, HPLC instrumentation, extraction, hydrolysis conditions, identification and quantification of Htyr and Tyr were according to Mastralexi et al. [10]. Elution conditions were as those described by IOC [9]. Elution was carried out on a 250 mm × 4.6 mm i.d., 5 μm Nucleosil 100, C18 column (MZ-Analysentechnik GmbH, Mainz, Germany).

#### 3.2.2. Laboratory 2

##### Protocol C

Chemicals and HPLC instrumentation were as described by Majetić-Germek et al. [34]. Elution using a Synergi 4 μm Hydro–RP 80 A (250 mm × 4.6 mm) column protected with security guard cartridge AQC 18 4 mm × 3.0 mm (both Phenomenex, Torrance, CA, USA), identification and quantification of individual phenols was according to the IOC method [9]. Tentative assignment of additional peaks to Htyr and Tyr derivatives was carried out using a LC–MS (TQd) equipment as described elsewhere Nenadis et al. [26]. The total Htyr and Tyr content were expressed as Tyr equivalents per 20 g oil.

#### 3.2.3. Laboratory 3

##### Protocol D

Chemicals, HPLC instrumentation, elution protocol using a Spherisorb ODS-1 (250 mm x 4.6 mm), with a particle size of 5 µm (Waters, Milford, MA, USA), identification and quantification of Htyr, Tyr, decarboxymethyl oleuropein aglycone, dialdehyde form, decarboxymethyl ligstroside aglycone, dialdehyde form, oleuropein aglycone and ligstroside aglycone were according to Esposto et al. [35]. The total Htyr and Tyr content expressed per 20 g oil was the sum of the above six compounds.

#### 3.2.4. Laboratory 4

##### Protocol E

Chemicals, HPLC instrumentation and Exactive–HCD (higher energy collisional dissociation) Orbitrap ESI^−^ (negative electrospray ionization) conditions were those described by Vichi et al. [36]. Elution conditions were as described by Nenadis et al. [26] on a Halo C18 fused-core column 2.1 mm × 100 mm. 2.7 μm particle size (Advanced Materials Technology, Wilmington, DE, USA).

The Htyr and Tyr derivatives evaluated in negative ionization mode setting the mass range to *m*/*z* 90–600 are shown in Table 7. 

The target compounds were quantified with respect to IS (*o*-coumaric acid) assuming that the response factor for all compounds relative to *o*-coumaric acid used as the internal standard was 1. The total Htyr and Tyr content expressed per 20 g oil was the sum of concentrations of the above compounds.

#### 3.2.5. Laboratory 5

##### Protocol F

^1^H-NMR measurements were carried out using a Bruker Avance-III HD 400 MHz spectrometer equipped with a 5 mm BBI probe (Bruker, Karlsruhe, Germany). 10 g of olive oil were extracted with 3 mL of extraction solvent (acetonitrile/acetonitrile-d_3_ (95/5, *v*/*v*) by shaking for 30 min (mechanical shaker) after which a clear phase separation was obtained by centrifugation (2 min at 4000 rpm) and 600 µL of the upper phase were directly transferred into the NMR tube. Acquisition was performed at 300 K with suppression of the large signal of non-deuterated acetonitrile employing the noesygppr1D pulse sequence. Thirty-two scans of 64 k complex data points were acquired with a spectral width of 20.5524 ppm. In addition to oleacein and oleocanthal, the different monoaldehydic and dialdehydic forms of oleuropein aglycone and ligstroside aglycone were assigned in analogy to the NMR data in the literature [37] and confirmed by a series of selective TOCSY experiments. For each individual derivative one of its aldehydic hydrogen atoms was used to determine the concentration. Using an external calibration, the concentrations of the respective compounds were obtained in mmol/L. As no differentiation was possible between Htyr and Tyr derivatives, the average of the respective molar masses was used to obtain results in mg/L (312 g/mol for oleacein/oleocanthal; 370 g/mol for the aglycone isomers) and the sum for all of the different derivatives was reported.

### 3.3. Statistical Analysis

Passing-Bablok regression analysis and Bland-Altman analysis were carried out with the aid of a website free of charge (https://bahar.shinyapps.io/method_compare/).

## 4. Conclusions

The EFSA health claim on ‘olive oil polyphenols’ raised many questions among scientists regarding the most appropriate analytical procedure that would combine accuracy with ease of application. The proposed fit for the purpose UHPLC approach combines these characteristics and it is also faster than HPLC procedures involving or not hydrolysis of the polar fraction.

## Figures and Tables

**Table 1:** Total (free and bound forms) Htyr and Tyr content (mg/20 g oil) of virgin olive oil (VOO) samples according to protocol A.

Samples	Total Htyr	Total Tyr	Sum *	Samples	Total Htyr	Total Tyr	Sum *
S-1	4.52 ± 0.46	5.70 ± 0.02	10	S-16	1.69 ± 0.03	0.00 ± 0.00	2
S-2	3.22 ± 0.01	2.86 ± 0.01	6	S-17	3.48 ± 0.08	5.93 ± 0.02	9
S-3	5.12 ± 0.03	4.13 ± 0.01	9	S-18	2.39 ± 0.02	2.00 ± 0.02	4
S-4	3.69 ± 0.02	4.64 ± 0.01	8	S-19	1.15 ± 0.01	0.45 ± 0.03	2
S-5	1.14 ± 0.01	1.54 ± 0.01	3	S-20	2.43 ± 0.02	3.21 ± 0.01	6
S-6	1.85 ± 0.02	3.45 ± 0.01	5	S-21	2.20 ± 0.02	1.61 ± 0.01	4
S-7	3.26 ± 0.07	3.90 ± 0.05	7	S-22	0.93 ± 0.01	1.59 ± 0.03	3
S-8	1.86 ± 0.01	1.89 ± 0.01	4	S-23	1.02 ± 0.01	1.16 ± 0.01	2
S-9	1.86 ± 0.01	1.26 ± 0.04	3	S-24	6.15 ± 0.10	3.73 ± 0.07	10
S-10	2.53 ± 0.01	2.10 ± 0.01	5	S-25	5.32 ± 0.03	2.27 ± 0.01	8
S-11	1.33 ± 0.01	1.82 ± 0.01	3	S-26	7.31 ± 0.02	4.61 ± 0.02	12
S-12	3.42 ± 0.01	1.85 ± 0.01	5	S-27	0.54 ± 0.00	1.42 ± 0.01	2
S-13	3.04 ± 1.01	2.24 ± 0.03	5	S-28	0.26 ± 0.04	0.10 ± 0.02	0
S-14	2.20 ± 0.02	1.60 ± 0.01	4	S-29	0.28 ± 0.11	0.44 ± 0.03	1
S-15	3.11 ± 0.32	5.29 ± 0.31	8	S-30	2.55 ± 0.01	4.23 ± 0.00	7

* Rounded to the first integer.

**Table 2:** Total (free and bound forms) Htyr and Tyr content (mg/20 g oil) of VOO samples according to protocol B.

Samples	Total Htyr	Total Tyr	Sum *	Samples	Total Htyr	Total Tyr	Sum *
S-1	5.30 ± 0.22	5.82 ± 0.05	11	S-16	0.68 ± 0.01	0.50 ± 0.01	1
S-2	1.38 ± 0.05	2.03 ± 0.03	3	S-17	2.29± 0.07	5.75 ± 0.19	8
S-3	3.57 ± 0.01	3.24 ± 0.04	7	S-18	2.49 ± 0.09	2.70 ± 0.17	5
S-4	2.53 ± 0.05	3.40 ± 0.11	6	S-19	1.11 ± 0.01	1.10 ± 0.02	2
S-5	1.80 ± 0.04	1.50 ± 0.04	3	S-20	2.24 ± 0.01	2.67 ± 0.03	5
S-6	2.50 ± 0.03	3.31 ± 0.06	6	S-21	1.50 ± 0.03	1.95 ± 0.13	3
S-7	2.97 ± 0.01	3.16 ± 0.11	6	S-22	0.17 ± 0.02	1.08 ± 0.02	1
S-8	1.42 ± 0.61	1.52 ± 0.02	3	S-23	0.62 ± 0.01	1.38 ± 0.02	2
S-9	2.05 ± 0.01	2.16 ± 0.12	4	S-24	6.12 ± 0.03	4.20 ± 0.08	10
S-10	1.43 ± 0.01	1.39 ± 0.04	3	S-25	4.55 ± 0.05	1.36 ± 0.03	6
S-11	1.49 ± 0.04	0.58 ± 0.02	2	S-26	5.71 ± 0.02	2.63 ± 0.06	8
S-12	2.18 ± 0.02	1.42 ± 0.02	4	S-27	2.98 ± 0.04	1.39 ± 0.02	4
S-13	2.10 ± 0.00	2.61 ± 0.01	5	S-28	0.16 ± 0.02	0.05 ± 0.02	0
S-14	2.04 ± 0.01	2.23 ± 0.01	4	S-29	0.19 ± 0.05	0.31 ± 0.03	0
S-15	3.80± 0.10	5.96± 0.09	10	S-30	1.42 ± 0.05	3.36 ± 0.05	5

* Rounded to the first integer.

**Table 3:** Total (free and bound forms) Htyr and Tyr content (mg/20 g oil) of VOO samples according to protocol C.

Samples	Total Htyr	Total Tyr	Sum *	Samples	Total Htyr	Total Tyr	Sum *
S-1	2.64 ± 0.01	1.99 ± 0.05	5	S-16	0.58 ± 0.00	1.03 ± 0.01	2
S-2	1.04 ± 0.03	1.36 ± 0.03	2	S-17	2.93 ± 0.12	2.61 ± 0.02	6
S-3	3.21 ± 0.03	2.02 ± 0.04	5	S-18	1.68 ± 0.01	1.25 ± 0.00	3
S-4	2.65 ± 0.09	2.31 ± 0.07	5	S-19	0.84 ± 0.00	1.21 ± 0.00	2
S-5	0.85 ± 0.05	0.90 ± 0.01	2	S-20	1.79 ± 0.01	1.48 ± 0.01	3
S-6	1.36 ± 0.05	1.82 ± 0.02	3	S-21	1.05 ± 0.04	1.04 ± 0.00	2
S-7	2.33 ± 0.02	1.69 ± 0.01	4	S-22	0.47 ± 0.02	1.28 ± 0.02	2
S-8	0.86 ± 0.01	0.82 ± 0.01	2	S-23	0.53 ± 0.03	0.83 ± 0.01	1
S-9	1.54 ± 0.07	1.10 ± 0.05	3	S-24	4.83 ± 0.05	1.99 ± 0.04	7
S-10	1.49 ± 0.01	0.99 ± 0.01	2	S-25	3.75 ± 0.01	1.29 ± 0.01	5
S-11	1.59 ± 0.01	1.27 ± 0.00	3	S-26	4.85 ± 0.02	1.86 ± 0.04	7
S-12	1.40 ± 0.05	0.85 ± 0.02	2	S-27	0.30 ± 0.03	1.13 ± 0.06	1
S-13	1.01 ± 0.04	1.02 ± 0.00	2	S-28	0.16 ± 0.00	0.80 ± 0.01	1
S-14	1.60 ± 0.03	1.28 ± 0.01	3	S-29	0.26 ± 0.01	0.77 ± 0.00	1
S-15	3.01 ± 0.01	2.76 ± 0.03	6	S-30	1.26 ± 0.02	1.93 ± 0.01	3

* Rounded to the first integer.

**Table 4:** Total (free and bound forms) Htyr and Tyr content (mg/20 g oil) of VOO samples according to protocol D.

Samples	Total Htyr	Total Tyr	Sum *	Samples	Total Htyr	Total Tyr	Sum *
S-1	12.67 ± 0.05	2.80 ± 0.02	15	S-16	2.18 ± 0.02	0.31 ± 0.00	2
S-2	4.00 ± 0.02	1.40 ± 0.00	5	S-17	8.51 ± 0.03	2.48 ± 0.02	11
S-3	10.81 ± 0.09	1.89 ± 0.01	13	S-18	5.97 ± 0.05	0.60 ± 0.01	7
S-4	7.47 ± 0.03	2.00 ± 0.02	9	S-19	2.81 ± 0.02	0.49 ± 0.00	3
S-5	3.38 ± 0.07	0.85 ± 0.01	4	S-20	4.88 ± 0.02	1.31 ± 0.00	6
S-6	5.43 ± 0.19	1.82 ± 0.02	7	S-21	2.95 ± 0.02	0.87 ± 0.00	4
S-7	8.53 ± 0.06	1.87 ± 0.02	10	S-22	1.07 ± 0.01	0.87 ± 0.00	2
S-8	4.58 ± 0.14	1.09 ± 0.01	6	S-23	1.47 ± 0.01	0.68 ± 0.00	2
S-9	4.99 ± 0.02	1.04 ± 0.01	6	S-24	15.40 ± 0.05	1.86 ± 0.01	17
S-10	5.26 ± 0.04	0.95 ± 0.01	6	S-25	11.58 ± 0.03	0.95 ± 0.02	13
S-11	5.01 ± 0.02	1.19 ± 0.01	6	S-26	16.77 ± 0.08	2.01 ± 0.02	19
S-12	5.34 ± 0.02	0.83 ± 0.01	6	S-27	1.00 ± 0.02	0.84 ± 0.00	2
S-13	4.11 ± 0.03	0.99 ± 0.01	5	S-28	0.57 ± 0.03	0.54 ± 0.02	1
S-14	6.16 ± 0.03	1.29 ± 0.01	7	S-29	1.12 ± 0.02	0.59 ± 0.00	2
S-15	8.71 ± 0.01	2.46 ± 0.01	11	S-30	5.42 ± 0.02	2.24 ± 0.02	8

* Rounded to the first integer.

**Table 5:** Total (free and bound forms) Htyr and Tyr content (mg/20 g oil) of VOO samples according to protocol E.

Samples	Total Htyr	Total Tyr	Sum *	Samples	Total Htyr	Total Tyr	Sum *
S-1	6.40 ± 0.04	3.38 ± 0.14	10	S-16	1.56 ± 0.07	0.57 ± 0.03	2
S-2	2.38 ± 0.10	1.51 ± 0.06	4	S-17	3.31 ± 0.01	2.01 ± 0.07	5
S-3	5.42 ± 0.15	1.87 ± 0.01	7	S-18	3.05 ± 0.29	1.67 ± 0.05	5
S-4	4.12 ±0.00	1.91 ± 0.00	6	S-19	1.44 ± 0.15	0.69 ± 0.06	2
S-5	1.70 ± 0.09	0.98 ± 0.03	3	S-20	1.89 ± 0.01	1.00 ± 0.01	3
S-6	2.37 ± 0.19	1.61 ± 0.06	4	S-21	0.82 ± 0.13	0.66 ± 0.03	1
S-7	4.38 ± 0.02	1.80 ± 0.06	6	S-22	0.47 ± 0.05	0.49 ± 0.02	1
S-8	2.34 ± 0.47	1.32 ± 0.06	4	S-23	0.64 ± 0.03	0.67 ± 0.02	1
S-9	2.90 ± 0.38	1.10 ± 0.05	4	S-24	6.41 ± 0.02	1.86 ± 0.01	8
S-10	2.77 ± 0.23	0.98 ± 0.09	4	S-25	3.89 ± 0.24	0.71 ± 0.02	5
S-11	2.54 ± 0.16	1.21 ± 0.14	4	S-26	6.50 ± 1.37	2.49 ± 0.06	9
S-12	3.09 ± 0.27	0.95 ± 0.06	4	S-27	0.53 ± 0.00	0.54 ± 0.02	1
S-13	1.48 ± 0.19	1.13 ± 0.12	3	S-28	0.22 ± 0.01	0.27 ± 0.01	0
S-14	3.66 ± 0.10	1.66 ± 0.19	5	S-29	0.30 ± 0.01	0.20 ± 0.01	0
S-15	6.08 ± 0.02	3.55 ± 0.12	10	S-30	3.18 ± 0.07	2.40 ± 0.07	6

* Rounded to the first integer.

**Table 6:** Total Htyr and Tyr (free and bound forms) content (mg/20 g oil) of VOO samples according to protocol F.

Samples	Sum *	Samples	Sum *
S-1	14	S-16	3
S-2	6	S-17	11
S-3	11	S-18	7
S-4	10	S-19	4
S-5	4	S-20	7
S-6	7	S-21	5
S-7	9	S-22	3
S-8	6	S-23	3
S-9	6	S-24	14
S-10	5	S-25	9
S-11	6	S-26	14
S-12	6	S-27	4
S-13	5	S-28	2
S-14	7	S-29	2
S-15	11	S-30	9

* Rounded to the first integer.

**Table 7:** Htyr and Tyr derivatives evaluated in negative ionization mode setting the mass range to *m*/*z* 90–600.

Theoretical *m*/*z*	Ion Elemental Formula	Compound
153.0552	[C_8_H_9_O_3_]^−^	Htyr
377.1242	[C_19_H_21_O_8_]^−^	Oleuropein aglycone isomers
319.1187	[C_17_H_19_O_6_]^−^	Decarboxymethyl oleuropein aglycone
393.1191	[C_19_H_21_O_9_]^−^	Oxygenated oleuropein aglycone isomers
335.1136	[C_17_H_19_O_7_]^−^	Oxygenated decarboxymethyl oleuropein aglycone
361.1293	[C_19_H_21_O_7_]^−^	Ligstroside aglycone isomers
303.1238	[C_17_H_19_O_5_]^−^	Decarboxymethyl ligstroside aglycone
319.1187	[C_17_H_19_O_6_]^−^	Oxygenated decarboxymethyl ligstroside aglycone

Tyr was not detected.

## Supplementary Materials

The supplementary materials are available online.

## Abbreviations

AOAC	Association of Analytical Communities
CD_3_CN	Deuterated acetonitrile
EFSA	European Food Safety Authority
EU	European union
GC-MS	Gas chromatography–mass spectrometry
HPLC	High performance liquid chromatography
HPLC-DAD	High performance liquid chromatography-diode array detection
Htyr	Hydroxytyrosol
IOC	International olive council
IS	Internal standard
LC-HESI-HRMS	Liquid chromatography- heated electrospray ionization-high resolution mass spectrometry
LC-HRMS	Liquid chromatography-high resolution mass spectrometry
LC-MS	Liquid chromatography- mass spectrometry
LC-MS(TQd)	Liquid chromatography-mass spectrometry (triple quadrupole)
NMR	Nuclear magnetic resonance spectroscopy
ODS	Octadecyl-silica
OO	Olive oil
PF	Polar fraction
TOCSY	Total correlation spectroscopy
Tyr	Tyrosol
UHPLC	Ultra-high performance liquid chromatography
UHPLC-DAD	Ultra-high performance liquid chromatography-diode array detection
VOO	Virgin olive oil
