# Supplementary material for: Toward a Harmonized and Standardized Protocol for the Determination of Total Hydroxytyrosol and Tyrosol Content in Virgin Olive Oil (VOO). The Pros of a Fit for the Purpose Ultra High Performance Liquid Chromatography (UHPLC) Procedure
Source: Molecules. 2019 Jul 2;24(13):2429. doi: 10.3390/molecules24132429 (PMC6651398; doi:10.3390/molecules24132429)
Supplement: Supplementary file 1 [file molecules-24-02429-s001.pdf]

**SUPPLEMENTARY MATERIAL**

**Table S1**. Total Htyr and Tyr (free and bound forms) content (mg/20 g oil) of VOO samples according to procotol C (C′) considering additional peaks assigned to Htyr or Tyr bound formsby LC-MS

| Samples | Total Htyr | Total Tyr | Sum | Samples | Total Htyr | Total Tyr | Sum * |
|---|---|---|---|---|---|---|---|
| S-1 | 3.81 ± 0.02 | 2.56 ± 0.01 | 6 | S-16 | 0.85 ± 0.00 | 1.25 ± 0.01 | 2 |
| S-2 | 1.24 ± 0.34 | 1.80 ± 0.03 | 3 | S-17 | 3.11 ± 0.24 | 3.01 ± 0.01 | 6 |
| S-3 | 4.00 ± 0.09 | 2.55 ± 0.04 | 7 | S-18 | 2.20 ± 0.00 | 1.62 ± 0.00 | 4 |
| S-4 | 2.77 ± 0.29 | 2.83 ± 0.08 | 6 | S-19 | 0.98 ± 0.19 | 1.40 ± 0.04 | 2 |
| S-5 | 1.36 ± 0.01 | 1.22 ± 0.02 | 3 | S-20 | 2.09 ± 0.01 | 1.95 ± 0.01 | 4 |
| S-6 | 1.78 ± 0.28 | 2.32 ± 0.00 | 4 | S-21 | 1.15 ± 0.17 | 1.51 ± 0.02 | 3 |
| S-7 | 2.69 ± 0.00 | 2.13 ± 0.00 | 5 | S-22 | 0.74 ± 0.04 | 1.66 ± 0.02 | 2 |
| S-8 | 1.02 ± 0.24 | 1.09 ± 0.02 | 2 | S-23 | 0.72 ± 0.19 | 1.14 ± 0.02 | 2 |
| S-9 | 1.91 ± 0.04 | 1.48 ± 0.05 | 3 | S-24 | 5.67 ± 0.07 | 2.28 ± 0.05 | 8 |
| S-10 | 1.60 ± 0.17 | 1.27 ± 0.02 | 3 | S-25 | 3.92 ± 0.29 | 1.48 ± 0.04 | 5 |
| S-11 | 2.00 ± 0.01 | 1.65 ± 0.00 | 4 | S-26 | 6.23 ± 0.02 | 2.32 ± 0.00 | 9 |
| S-12 | 1.60 ± 0.30 | 1.04 ± 0.04 | 3 | S-27 | 0.34 ± 0.06 | 1.35 ± 0.15 | 2 |
| S-13 | 1.38 ± 0.03 | 1.38 ± 0.00 | 3 | S-28 | 0.22 ± 0.10 | 0.98 ± 0.03 | 1 |
| S-14 | 2.10 ± 0.04 | 1.68 ± 0.02 | 4 | S-29 | 0.47 ± 0.01 | 1.04 ± 0.00 | 1 |
| S-15 | 3.21 ± 0.34 | 3.17 ± 0.06 | 6 | S-30 | 1.41 ± 0.24 | 2.23 ± 0.03 | 4 |

*rounded to the first integer; values ≥5 mg/20 oil are highlighted in grey

**Table S2**. Estimate (EST), standard error (SE) and lower-upper confidence interval (LCI-UCI) for intercept and slope according to Passing Bablok regression analysis applied pairwise for the rounded values of protocol A (reference method) with those of B-E

| Protocols | | EST | SE | LCI* | UCI* |
|---|---|---|---|---|---|
| A-B | Intercept | 0.1348 | 0.4976 | -0.8845 | 1.154 |
| | Slope | 0.8469 | 0.08275 | 0.6774 | 1.016 |
| A-C | Intercept | 0.3682 | 0.2875 | -0.2208 | 0.9572 |
| | Slope | 0.5347 | 0.04781 | 0.4368 | 0.6327 |
| A-C′ | Intercept | 0.6471 | 0.2708 | 0.09248 | 1.202 |
| | Slope | 0.6216 | 0.04503 | 0.5293 | 0.7138 |
| A-D | Intercept | -0.4803 | 0.5936 | -1.696 | 0.7356 |
| | Slope | 1.461 | 0.09871 | 1.259 | 1.663 |

| | | | | | |
|---|---|---|---|---|---|
| A-E | Intercept | 0.0929 | 0.4977 | -0.9267 | 1.112 |
| | Slope | 0.7912 | 0.08277 | 0.6216 | 0.9607 |

*green color indicates that the criterion is satisfied

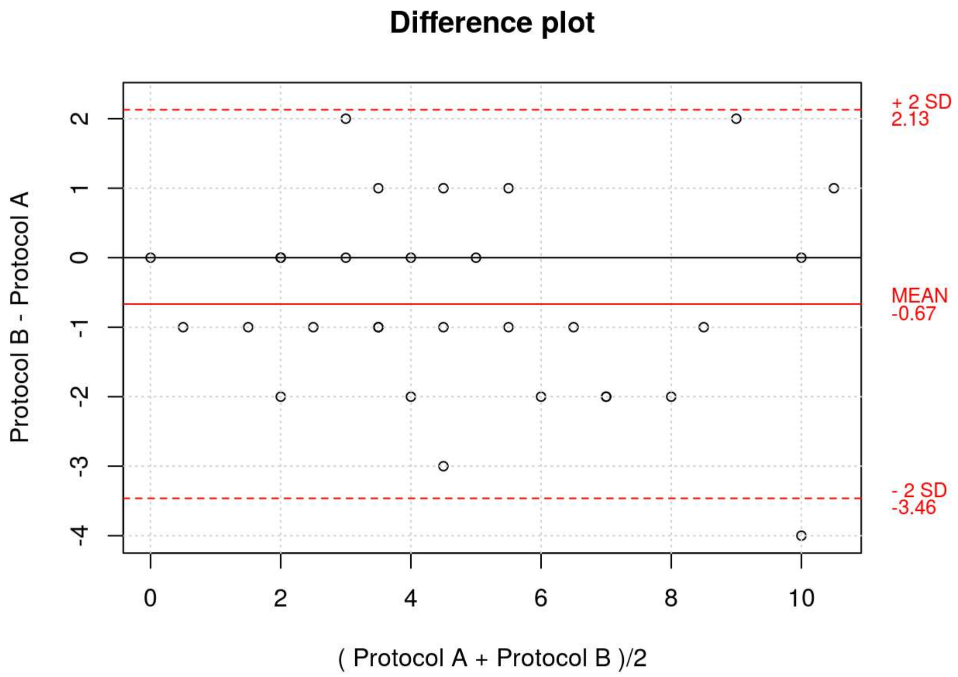


**Figure S1**. Difference plot of protocol A and B rounded values according to Bland-Altman analysis

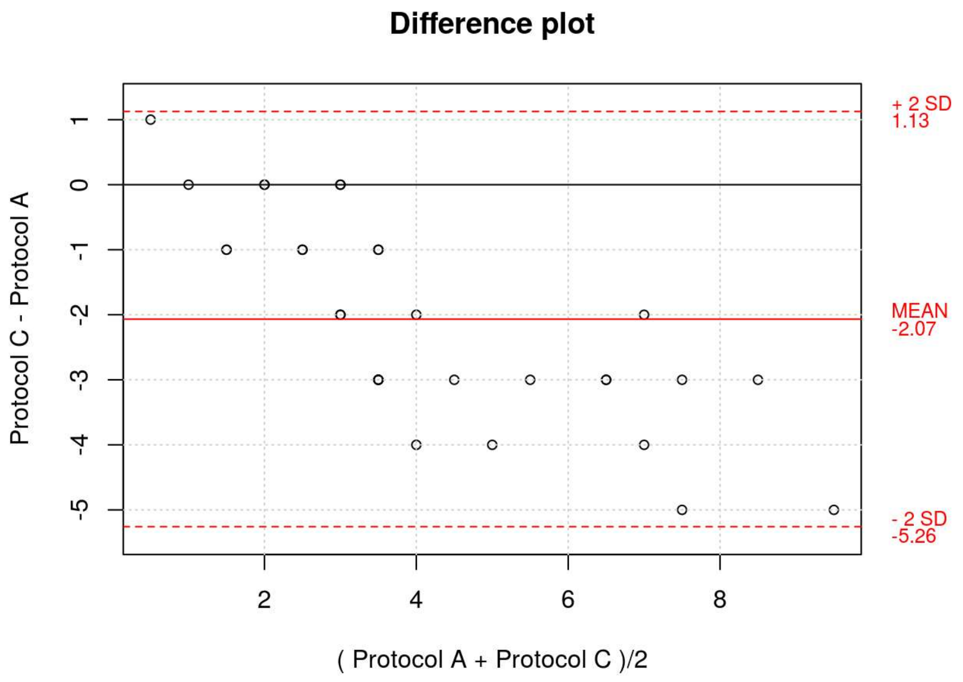


**Figure S2**. Difference plot of protocol A and C rounded values according to Bland-Altman analysis

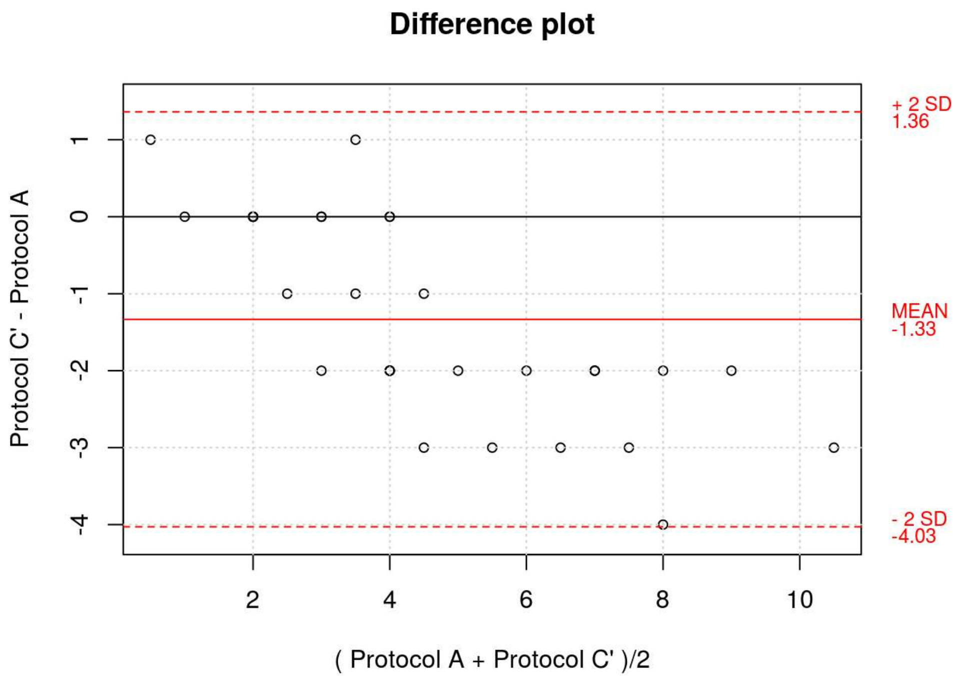


**Figure S3**. Difference plot of protocol A and C′ rounded values according to Bland-Altman analysis

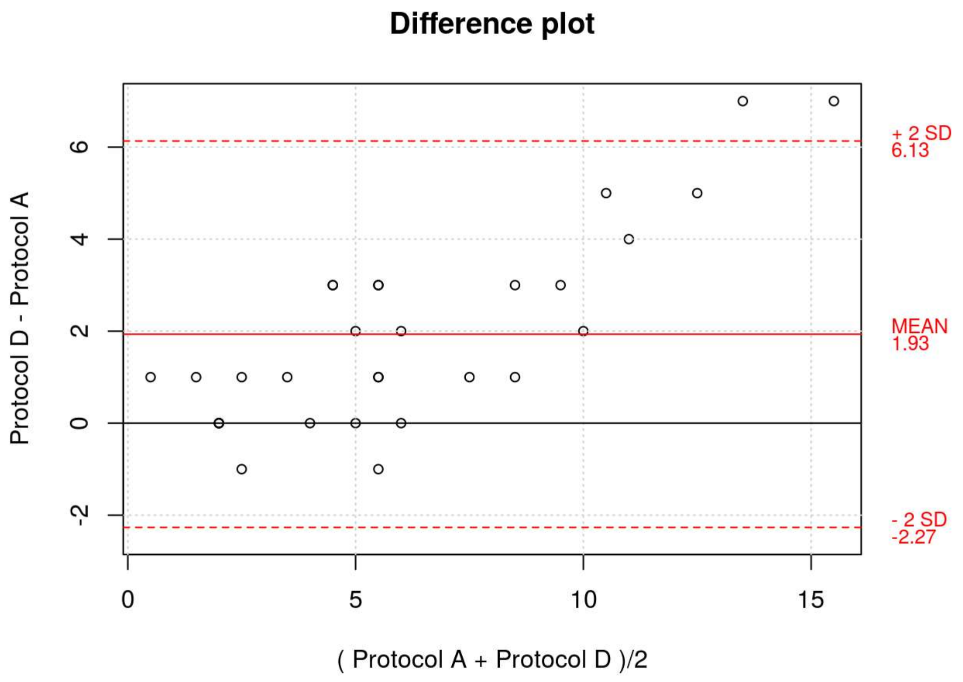


**Figure S4**. Difference plot of protocol A and D rounded values according to Bland-Altman analysis

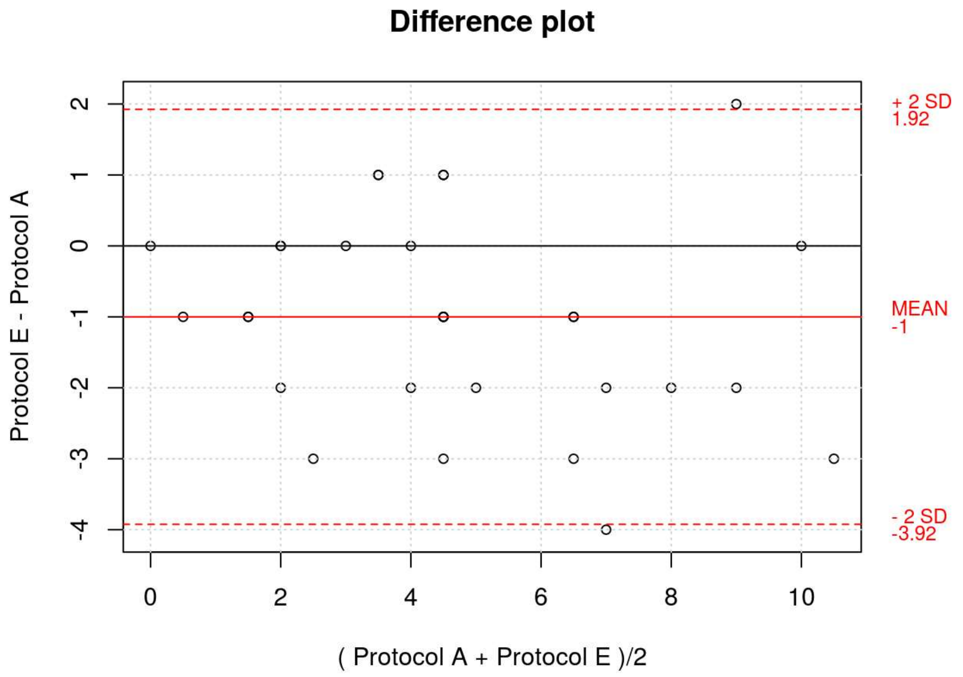


**Figure S5**. Difference plot of protocol A and E rounded values according to Bland-Altman analysis

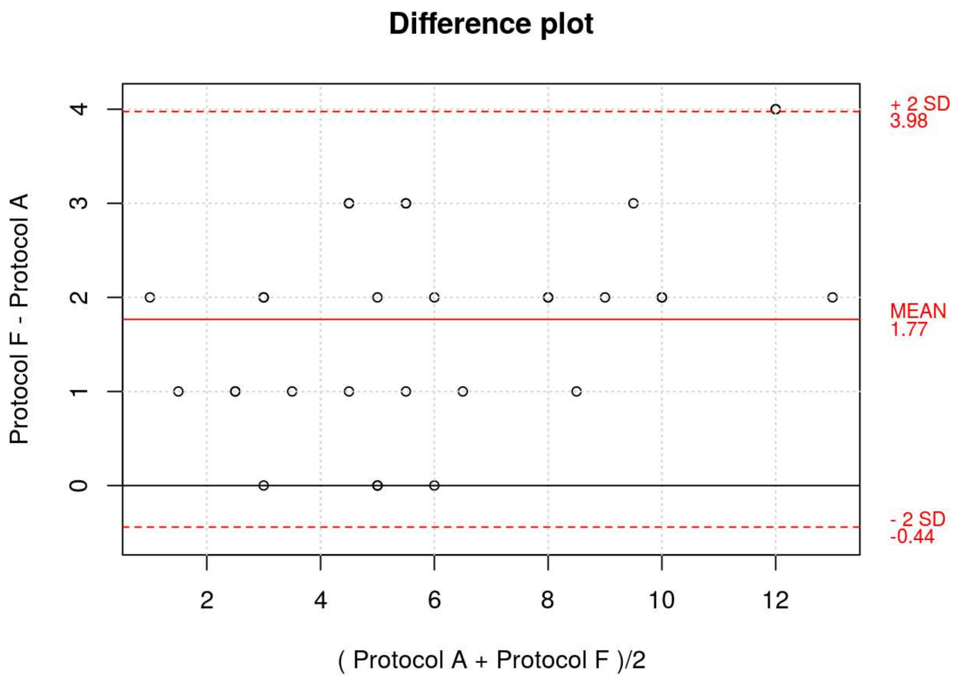


**Figure S6**. Difference plot of protocol A and F rounded values according to Bland-Altman analysis
